# Measurement and determinants of multidimensional urban poverty: Evidence from Shandong Province, China

**DOI:** 10.1371/journal.pone.0300263

**Published:** 2024-05-17

**Authors:** Bo Zhao, Phaik Kin Cheah, Priscilla Moses

**Affiliations:** 1 Faculty of Arts & Social Science, Universiti Tunku Abdul Rahman, Kampar, Perak, Malaysia; 2 College of Politics and Law, Heze University, Heze, Shandong, China; 3 Faculty of Creative Industries, Universiti Tunku Abdul Rahman, Kajang, Selangor, Malaysia; University of Sargodha, PAKISTAN

## Abstract

China eliminated rural poverty under current poverty standards in 2020. However, compared with rural poverty, urban poverty in China has been somewhat neglected. This paper aims to discover the changes and determinants of multidimensional urban poverty in Shandong Province, a representative province in Eastern China. Using a nationally representative panel dataset, the China Family Panel Studies, and the Dual Cutoff method, this study creates a multidimensional poverty index with four dimensions and 11 indicators to measure urban poverty in Shandong Province. This paper discovers that while the incidence of multidimensional urban poverty in Shandong Province decreased from 47.62% in 2010 to 36.45% in 2018, the intensity of multidimensional poverty only decreased from 41.27% to 37.25%, which indicates the inadequacy of urban anti-poverty efforts in Shandong Province. This paper also uses logistic regression to identify the determinants of multidimensional urban poverty. The findings suggest that income, health, drinking water, and durable goods are the main determinants of multidimensional urban poverty in Shandong Province. Based on these findings, this study provides targeted recommendations for future urban anti-poverty policies in Shandong Province.

## 1. Introduction

### 1.1. Research background

#### 1.1.1. Urban poverty situation in China

Since initiating economic reforms in 1978, China has significantly reduced its poverty levels [1–3]. The poverty rate decreased from 84% in 1981 to 0.1% in 2019, measured against the World Bank’s standard of living of less than USD 1.9 per day [4, 5]. In 2020, China declared the elimination of rural poverty, adhering to its contemporary poverty benchmarks of an annual income of 2300 CNY (338 USD) per capita (2010 constant prices), translating to approximately 2.20 USD per day in terms of purchasing power parity [6]. This milestone, however, primarily addresses absolute poverty; The challenge of relative poverty remains significant [7]. Notably, China’s poverty thresholds are specific to rural areas, lacking a unified standard for urban areas. Local governments independently establish minimum living allowances, leading to varied standards [8]. Urban poverty has not received the same level of targeted intervention as rural poverty [3], suggesting a degree of oversight in urban poverty alleviation efforts [9]. With the rural poverty issue largely addressed under current benchmarks, urban poverty emerges as an increasingly critical and pressing issue [10–13].

China’s poverty metrics predominantly focus on absolute poverty, aiming to provide for basic health needs and essential expenditures including clothing, utilities, education, and healthcare [14, 15]. These measures are primarily monetary, focusing on income or expenditure. However, the complexity of poverty transcends mere financial parameters [15, 16]. According to Nobel laureate economist Sen [17–19], poverty encompasses capability deprivation or lack thereof. The United Nations Development Programme (UNDP) [20, 21] and the World Bank [22] conceptualize poverty as the denial of choices and opportunities, encompassing not just material shortages like food and shelter, but also the lack of access to education, healthcare, clean water, and sanitation. Additionally, it involves issues of insecurity, social and institutional exclusion, and vulnerability to various adverse circumstances including violence. This multifaceted nature of poverty necessitates a multidimensional approach to its measurement [23, 24].

#### 1.1.2. The development of urban poverty in China

Prior to 1978, during the era of China’s planned economy, poverty was predominantly a rural phenomenon [9, 25–27]. This disparity stemmed from a rigid separation between urban and rural economies. Urban residents, during this period, were provided with food rations and enjoyed the benefits of full employment, including job security. Most urban families earned a modest, yet stable income [26]. Additionally, urban workers were recipients of comprehensive welfare benefits like health insurance, pensions, and maternity compensation, all of which were provided by their employers [28, 29]. The landscape of urban poverty in China began to change with the market-oriented reforms and the restructuring of state-owned enterprises (SOEs) in the 1990s [30]. These reforms led to a significant upheaval in the economic structure. Over 73% of China’s SOEs were dismantled, and more than 42% of SOE employees were laid off, resulting in the loss of their primary means of subsistence [31]. Concurrently, the occupation-based welfare system, a legacy of the planned economy era, began to diminish. The emerging social security system, including the minimum living allowance, was not yet fully established. This transitional phase contributed to the intensification of urban poverty in China [9, 32, 33].

#### 1.1.3. The characteristics of urban poverty in China

In contemporary China, urban incomes are notably higher than those in rural areas. In 2021, the income of the lowest 20% of urban residents was recorded at CNY 16,745, in stark contrast to the CNY 4,855 earned by their rural counterparts [34]. This disparity indicates that the income of the lowest urban demographic significantly exceeds the rural absolute poverty threshold of approximately CNY 4000 [35]. Consequently, it can be inferred that absolute poverty in urban regions is substantially less prevalent compared to rural areas. Urban poverty in China is primarily characterized by relative poverty [11, 36, 37], defined as the inadequacy of resources to maintain a standard of living that is either accustomed to or widely recognized in their societal context [37, 38]. The relative deprivation experienced by the urban poor is more acute compared to rural populations, often leading to heightened frustration and anxiety. These factors can exacerbate social tensions and impact societal stability. Assessing urban poverty is vital for the government to develop effective urban poverty alleviation policies aimed at fostering social harmony and resolving social conflicts [39].

Addressing urban poverty is also integral to realizing the Sustainable Development Goals (SDGs) set by the United Nations, as well as China’s aspiration for widespread prosperity. The primary goal of the SDGs is to eliminate extreme poverty universally and reduce poverty in all its forms by at least half by 2030. Furthermore, China aims to make substantial progress toward achieving common prosperity for all by 2035, with a notable reduction in the disparities in living standards among its citizens [40]. These objectives are unattainable without effectively tackling the issue of urban poverty.

### 1.2. Research purpose

The primary aim of this research is to explore strategies for mitigating multidimensional urban poverty in Shandong Province. The study is structured around three key objectives. The first objective is to assess the extent of multidimensional urban poverty in Shandong Province. This involves a comprehensive measurement of poverty that transcends mere economic parameters to include aspects like access to education, healthcare, and living standards. The second objective is to identify the underlying causes of this multifaceted urban poverty within the province. Understanding these root causes is crucial for developing effective interventions. The third and final objective is to formulate actionable recommendations for alleviating multidimensional urban poverty in Shandong Province in the future. These recommendations will be aimed at addressing both the symptoms and root causes of poverty, thereby contributing to the broader goal of enhancing social welfare and stability in the region.

### 1.3. Research gaps

The examination of existing literature reveals several deficiencies in the measurement of multidimensional poverty. These include debates over the selection of dimensions and indicators, as well as the determination of appropriate weights and poverty thresholds [12, 41–44]. The Global Multidimensional Poverty Index (MPI), developed by the UNDP, stands as one of the most recognized measures for assessing multidimensional poverty [45]. However, many researchers have merely replicated the Global MPI with minor adjustments to its dimensions and indicators, leading to a certain degree of arbitrariness in these studies [46]. This research aims to refine current measurement methodologies by tailoring indicators to better reflect the specific conditions in Shandong Province and modifying the poverty cutoff to align more closely with the Global MPI’s foundational logic [21]. Additionally, this study incorporates techniques such as dominance analysis, correlation tests, and multicollinearity tests to assess the robustness of indicators, weights, and poverty cutoffs in measuring multidimensional poverty, thereby enhancing the reliability of the findings.

Another gap in the literature is the limited examination of poverty’s causes from diverse perspectives, including individual, household, community, and regional levels [47, 48]. This study addresses this gap by integrating individual, household, and environmental indicators in a regression model. This model will analyze and discuss various causes of urban poverty, such as characteristics of the household head, household size and composition, household assets, and access to infrastructure.

Furthermore, research on poverty in China has predominantly focused on rural poverty, with urban poverty receiving comparatively less attention [9, 25–27]. Additionally, China’s primary approach to measuring poverty still relies on monetary dimensions to assess basic human needs [49]. Given the eradication of absolute poverty under the current standards in China by 2020, this study’s focus on multidimensional poverty is timely and pertinent. It contributes to the development of a new, comprehensive urban poverty standard in China, reflecting the evolving nature of poverty in the urban context [10, 12, 49].

### 1.4. Research significance

This study makes significant contributions to the field of urban poverty research through several key advancements. Firstly, it enhances existing methods for measuring multidimensional poverty. This enhancement includes refining the settings of poverty thresholds and weights and implementing a series of robustness tests. These improvements aim to increase the accuracy in identifying urban poor households.

Secondly, the research provides a detailed analysis of the evolution of multidimensional urban poverty in Shandong Province. It examines changes in both the incidence and intensity of poverty, revealing that the reduction in urban poverty in the province has been primarily due to a decrease in poverty incidence rather than a reduction in poverty intensity.

Thirdly, this study employs logistic regression to investigate the multifaceted nature of urban poverty in Shandong Province. This analysis identifies the primary factors contributing to multidimensional urban poverty within the region, offering valuable insights into the underlying causes of poverty.

Finally, the paper discusses the findings of the multidimensional urban poverty study in Shandong Province and leverages these insights to propose targeted recommendations. These recommendations are aimed at informing and enhancing future urban anti-poverty policies in Shandong Province. Through these contributions, the study not only addresses a critical gap in urban poverty research but also provides practical guidance for policymakers and stakeholders in the region.

### 1.5. Shandong Province

Shandong Province, a key economic region, is located on the Northeastern coast of China. Geographically positioned on the Eastern periphery of the North China Plain and along the lower reaches of the Yellow River, it borders the Bohai Sea to the north and the Yellow Sea to the South. Its strategic location offers a unique coastal interface with the Korean peninsula [50]. The province has a substantial population of 101.65 million and covers an area of 157,100 square kilometers [51].

In terms of economic development, Shandong’s GDP per capita in 2021 was USD 12,668, slightly exceeding the national average for China [52]. However, the per capita disposable income and consumption expenditure of urban residents in Shandong were slightly below the national average at CNY 47,066 (USD 6921) and CNY 29,314 (USD 4310), respectively [53, 54]. The economic structure mirrors that of China, with a dominance of secondary industries and a relatively less developed service sector [55]. Currently, Shandong is undergoing a significant economic transition, moving from traditional growth drivers to new ones, a process that could potentially intensify urban poverty [56, 57].

Demographically, according to the Shandong Province Statistical Yearbook 2021 [52] and the Bulletin of the 7th National Population Census of Shandong Province [58], Shandong exhibits diverse characteristics. The average household size stands at 2.7, with a gender ratio of approximately 50.8 males to 49.2 females. The population distribution includes 18.4% aged 0–14 years, 65.7% aged 15–64 years, and 15.9% aged 65 years and older. The youth dependency ratio is 17.48%, and the aged-dependency ratio is 15.42%. The average housing area per capita is 39.9 square meters. Educational attainment varies, with 14.38% holding college degrees, 14.39% having upper secondary degrees, 35.78% with lower secondary, and 23.69% with elementary degrees. The population aged 15 and older has an average of 9.75 years of education, and the illiteracy rate stands at 3.26%. The Han Chinese form the majority of the population at 99.11%, with ethnic minorities, primarily Hui, constituting 0.89% [52, 58].

## 2. Literature review

### 2.1. The measurement of poverty

The scholarly exploration of poverty began with livelihood surveys by Charles Booth in 1886 and Seebohm Rowntree in 1899. Rowntree’s research in York led to the concept of absolute poverty, defined as the minimum income necessary for physical subsistence [27, 59]. Rowntree employed the standard budget approach, which encompasses essential items like food, clothing, housing, and fuel to establish the poverty line [27]. This standard budget approach, however, primarily focuses on basic survival needs and overlooks higher-level necessities such as education, healthcare, and social security. With the eradication of absolute poverty in some countries, like China, the relevance of this concept has diminished.

Peter Townsend expanded the poverty discourse by introducing the concept of relative deprivation [22, 38, 60]. Townsend’s theory encompasses a broad spectrum of living standards, including diet, housing conditions, health, education, and social relations [38]. However, this approach has been criticized for equating poverty with inequality [61, 62]. For instance, China’s higher income inequality post-economic reform does not necessarily imply higher poverty compared to the pre-reform era, despite relative poverty measures suggesting otherwise [63]. The relative poverty measure evaluates various aspects of living standards in monetary terms, setting a poverty line based on a percentage (such as 60%) of the average or median income [64]. This approach has been adopted by the European Union and the Organisation for Economic Co-operation and Development (OECD) [65, 66].

**Fig 1:**
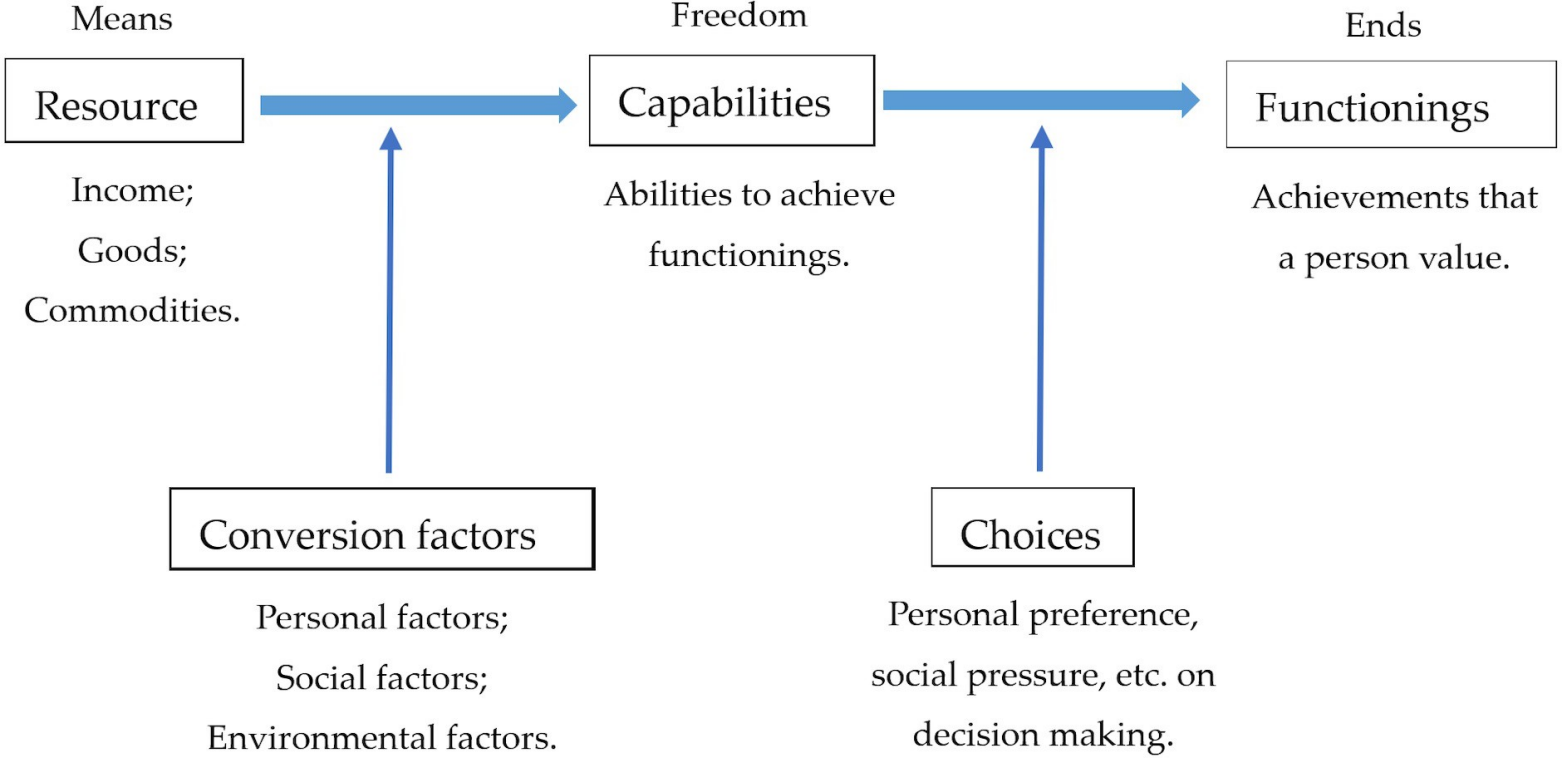
The framework of the capability approach. Functionings denote the achievements valued by individuals. Capabilities refer to the freedom and opportunities for individuals to achieve certain functionings. Resources serve as the means to achieve valued ends. These resources are transformed into functionings through capabilities. The arrows depict the direction of transformation.

Sen proposed the capability approach and paved the way for multidimensional poverty research [17–19]. The capability approach is a conceptual framework for the assessment of an individual’s well-being. The capability approach consists of three key concepts: functionings, capabilities, and resources (see Fig 1) [67–69]. Among these, functionings denote the achievements that an individual values, such as being well-nourished, having shelter, being able to receive education, and participating in community social activities [67, 69]. Capabilities refer to the abilities that enable a person to achieve or choose certain functionings they value [67, 69]. While functionings are the “beings and doings” of a person, capabilities are what people are able to do and be in their lives [68]. According to Sen [19], resources, such as income, goods, and commodities, are only means to well-being and not an end in itself. The relationship between resources and capabilities is mediated by the “conversion factors”; they help convert a set of resources into capabilities and capabilities into functionings [67, 70]. Conversion factors include three types: personal conversion factors, such as physical condition, gender, and skills; social conversion factors, such as public policies and social norms; and environmental conversion factors, which emerge from the physical or built environment in which a person lives [68].

Building on Sen’s framework, researchers developed various multidimensional poverty measures. The Fuzzy Set method by Cerioli and Zani [71] and the Totally Fuzzy and Relative method by Cheli and Lemmi [72] aimed to reduce arbitrariness in setting poverty thresholds. However, they have the drawback of being unable to be disaggregated by population subgroups [73, 74]. The Foster–Greer–Thorbecke (FGT) method by Foster et al. [75] primarily focuses on the depth of poverty. The Dual Cutoff method proposed by Alkire and Foster has gained widespread recognition and is being adopted by the UNDP [76, 77]. The advantage of this method lies in its flexibility, allowing adaptation to different dimensions and indicators, facilitating disaggregation by poverty dimensions or population subgroups, thus providing a more nuanced understanding of the factors leading to multidimensional poverty [78, 79]. Therefore, this study has chosen this method.

### 2.2. Theoretical framework and hypothesis development

This research constructs its theoretical framework grounded in Sen’s capability approach, focusing on three primary independent variables: resources, capabilities, and conversion factors:

1. Resources
  Income, while not a comprehensive measure of poverty, remains a critical means to access valuable ends and is thus included in the logit model, supported by research from Wang [8], the World Bank [22], and others [47, 80, 81]. Unemployment, a major contributor to urban poverty emergence in China [8, 25, 26], is also considered a significant variable. Additionally, household assets, recognized as a crucial component of resources in Sen’s framework [19, 68], are incorporated into the model.
2. Capabilities
  Capabilities represent the abilities that enable an individual to achieve valued functionings. Health and education are critical in expanding capabilities, empowering individuals to achieve valuable functionings [18]. With 40% of registered poor households in China falling into poverty due to illness [82], and a significant proportion of poor household heads having low education levels [83], variables such as education level, health status of household members, and medical insurance coverage are integral to the model. These choices are reinforced by findings from multiple studies [79, 84–86].
3. Conversion Factors
  This includes personal, social, and environmental factors. Personal factors, intrinsic to individuals, are represented in the model through characteristics like the household head’s age, gender, and marital status [68]. Social factors, reflecting the household as the basic social unit [87], are captured through variables like household size and dependency ratio. Environmental factors, encompassing aspects like climate, infrastructure, and accessibility, are addressed through variables such as access to clean drinking water and cooking fuel [68].

The conceptual framework developed in this study aims to identify the determinants of multidimensional urban poverty in Shandong Province. By incorporating these variables into a logit model, the study seeks to provide a comprehensive understanding of the factors influencing urban poverty in the region, aligning with the multidimensional perspective of poverty measurement (see Table 1). This approach enables the identification of targeted interventions for poverty alleviation in the context of Shandong Province.

**Table 1:** The conceptual framework for logistic regression.

Capability Approach Concepts	Concepts in the Logit Model		Variables in the Logit Model
**Resources**	Income		Income per capita
	Employment		Employed
			Unemployed
	Asset ownership		Car ownership
			Homeownership
			Living area per capita
			Durable good ownership
	Government subsidy		Government subsidized
**Capabilities**	Education		No education
			Elementary education
			Lower secondary education
			Upper secondary education
	Health		Household member unhealthy
			All household members have medical insurance
**Conversion factors**	Personal factors	Individual characteristics	Marital status
			Gender
			Age
	Social factors	Household structure	Aged-dependency ratio
			Youth dependency ratio
			Household size
	Environmental factors	Infrastructure accessibility	Household has clean drinking water
			Household has clean cooking fuel

Based on the aforementioned theory and our understanding of Shandong Province, China, the following hypotheses were developed for this study:

**Hypothesis 1**: Households with more income are less likely to fall into poverty. This hypothesis is grounded in the understanding that income, while not a comprehensive indicator of poverty, remains a crucial means to accessing valuable ends and enhancing capabilities. According to Sen’s capability approach, income, along with commodities and assets, form fundamental components of resources [67–69]. These resources play a significant role in enhancing an individual’s or household’s capabilities. In the context of Shandong Province, where economic development and income levels are reflective of national averages, this hypothesis assumes relevance. Higher income levels are likely to provide households with better access to essential resources, thereby reducing their likelihood of falling into poverty. This assumption is consistent with the broader understanding in poverty research that income, despite its limitations as a sole measure, is an important determinant in the alleviation of poverty conditions. The verification of this hypothesis would contribute to a nuanced understanding of the role of income in mitigating poverty within the urban settings of Shandong Province.
**Hypothesis 2**: Households in which all household members are healthy and have medical insurance are less likely to fall into poverty. Health is recognized as a critical expansion of capability, enhancing individuals’ freedom and opportunities to engage in valuable functionings [18, 68, 88]. Second, health is a vital component of human capital [80]. Empirical studies have shown that improving health can significantly contribute to poverty reduction [23, 47, 79, 80, 86]. In the context of Shandong Province, where healthcare access and quality may vary, this hypothesis is particularly relevant. It underscores the importance of health and medical insurance as key factors in mitigating the risk of poverty.
**Hypothesis 3**: Household heads with higher education levels are less likely to fall into poverty. Education is considered a crucial expansion of capability within Sen’s framework, empowering individuals to acquire knowledge and skills that enhance income and productivity [68]. As a form of human capital, education plays a significant role in improving economic outcomes [12, 80, 86]. In an urban context like Shandong Province, where educational opportunities and outcomes can significantly impact economic and social mobility, this hypothesis holds particular importance. It aims to establish the link between the educational attainment of household heads and the likelihood of poverty, highlighting education as a key tool in poverty alleviation efforts.
**Hypothesis 4**: Households with large sizes or high dependency ratios are more likely to fall into poverty. This hypothesis is grounded in the recognition of household characteristics as crucial conversion factors within Sen’s capability approach [89]. Empirical studies indicate that an increase in household size or dependency ratio tends to elevate household expenditures, thereby elevating the risk of poverty [23, 47, 79]. This hypothesis aims to explore the impact of household demographics on economic stability in Shandong Province.
**Hypothesis 5**: Households without access to clean drinking water or cooking fuel are more likely to fall into poverty. In the capability approach framework, infrastructure elements are regarded as significant environmental conversion factors [67–69]. The necessity of clean drinking water and cooking fuel for maintaining a high standard of living is underscored in the Global MPI of the UNDP [20, 21]. This hypothesis seeks to investigate the correlation between access to basic infrastructural amenities and poverty in urban areas of Shandong Province.
**Hypothesis 6**: Households without housing or possessing no more than one durable good are more likely to fall into poverty. Within Sen’s capability approach, assets are considered essential resources that reflect a household’s long-term material well-being and are instrumental in achieving valuable ends [67–69, 79]. This hypothesis is intended to examine the role of material assets in influencing the economic conditions of households in Shandong Province, assessing their contribution to poverty alleviation or exacerbation.

## 3. Data and methodology

### 3.1. Data sources

This paper measured multidimensional urban poverty in Shandong Province, China, using a nationally representative, annual longitudinal dataset of Chinese communities, families, and individuals, the China Family Panel Studies (CFPS), conducted by the Institute of Social Science Survey of Peking University, China [90]. The CFPS collected economic, as well as non-economic, well-being data on the economic activity, educational outcomes, family dynamics and relationships, and health of the Chinese population [90]. Initiated with a baseline survey in 2010, the CFPS has conducted subsequent surveys in 2012, 2014, 2016, and 2018 [90]. Employing a multistage probability sampling approach, it selected samples in three phases: county/district, village/neighborhood community, and household, thereby covering 16,000 households across 25 provinces, including Shandong Province.

This paper used data from CFPS in Shandong Province for 2010, 2012, 2014, 2016, and 2018 (see S1–S5 Datasets). Because this paper focused on urban areas, samples from rural areas and samples with missing data were excluded. After data cleaning, the remaining sample sizes were 252, 248, 240, 293, and 332, respectively. According to Cochran’s [91] formula, if the confidence level is set to 95% and the response distribution is set to 50%, the margin of error calculated using Cochran’s formula will be 6.17, 6.22, 6.33, 5.73, and 5.38, respectively. While margins of error in social science research typically range between 3% and 5%, the National Institutes of Health in the United States acknowledges a broader range of 3% to 7% as acceptable in such research [92], validating the margins applied in this study.

The CFPS database encompassed both household and individual data. Household data pertained to household income and living standards, while individual data focused on the education and health of household members. This study integrated individual data with household data, analyzing it with the household as the foundational unit [93, 94].

### 3.2. Measuring multidimensional poverty

This study employed the Dual Cutoff method, as proposed by Alkire and Foster [78], to measure multidimensional poverty. The methodology involves several steps:

This study first built a *n*×*d* matrix *X*, where *n* denotes the households to be measured, and *d* denotes the dimensions used for poverty measurement. The element *x*_*ij*_ denotes the deprivation score of household *i* in dimension *j*. Higher deprivation scores mean higher poverty intensity [60]. This paper set a d-dimensional vector *Z* = (*z*_1_,…,*z*_*d*_), where *z*_*j*_ is the cutoff for the poverty threshold in dimension *j*. If *x*_*ij*_ ≥ *z*_*j*_, then household *i* is considered poor in dimension *j*.

A deprivation matrix *g*^0^ is constructed where $g_{ij}^{0}$ denotes the deprivation score of household *i* in dimension *j*. And in matrix *g*^0^, if *x*_*ij*_ ≥ *z*_*j*_, $g_{ij}^{0}=1$; if not, $g_{ij}^{0}=0$. We set a d-dimensional vector *w* = (*w*_1_,…,*w_d_*), where *w*_*j*_ denotes the weight of dimension *j*. The deprivation score of household *i*, denoted by *c*_*i*_, equals $\sum_{i=1}^{d}w_{j}g_{ij}^{0}$.

A poverty cutoff *k* was set to identify multidimensional poverty; if *c*_*i*_ ≥ *k*, then household *i* is identified as multidimensionally poor. This paper calculated the multidimensional poverty incidence, denoted by *H*, by dividing the number of multidimensional poor by the total number of respondents [76]. The formula is $H=\frac{q}{n}$, where *q* denotes the number of multidimensional poor, and *n* denotes the total number of respondents.

A poverty cutoff *k* was set to identify multidimensional poverty; if *c*_*i*_ ≥ *k*, then household *i* was identified as multidimensionally poor. This paper calculated the multidimensional poverty incidence, denoted by *H*, by dividing the number of multidimensional poor by the total number of respondents [76]. The formula is $H=\frac{q}{n}$, where *q* denotes the number of multidimensional poor, and *n* denotes the total number of respondents.

The average deprivation score (*A*) was calculated by dividing the sum of the deprivation score of the multidimensional poor household by the total number of poor households [76]. The formula is $A=\frac{\sum_{i=1}^{n}c_{i}(k)}{q}$. The adjusted headcount ratio (*M*_0_), which reflected the incidence and intensity of multidimensional poverty, is calculated by *H* times *A* [77, 93]. The formula is *M*_0_ = *H* × *A*.

According to Alkire and Foster [70], *M*_0_ can be disaggregated by indicators to determine the contributions of indicators to multidimensional poverty. The formula of the contribution of dimension *j* to overall multidimensional poverty is $M_{0j}=\frac{w_{j}CH_{j}}{Mo}*100$, where *w*_*j*_ denotes the weight of indicator *j*, and *CH*_*j*_ denotes the censored headcount ratio of indicator *j*, which means the proportion of people who are both multidimensionally poor and deprived in that indicator [78].

### 3.3. Dimensions and indicators

The selection of dimensions and indicators is critical in the measurement of multidimensional poverty, as it directly influences the identification of the poor [94]. In choosing these dimensions and indicators, it is important to consider regional variations in living standards, cultural characteristics, and consumption habits [95]. This study adapted the Global MPI of the UNDP to the context of China, with specific attention to the average living conditions in Shandong Province [23, 94]. The Global MPI of the UNDP measures poverty in three dimensions: health (nutrition and child mortality), education (years of schooling and school attendance), and living standards (cooking fuel, sanitation, drinking water, electricity, housing, and assets) [94]. In this study, modifications were made to the Global MPI’s dimensions to align with available data and regional specifics: (1) Health dimension–due to the lack of data on child mortality in the CFPS, this indicator was removed. Instead, self-rated health status was included, considering its strong correlation with other health metrics, such as mortality rates [96–98]. Additionally, medical insurance was incorporated as an indicator, reflecting research that links increased access to medical insurance with improved household health outcomes [47, 84, 99]. (2) Standard of living dimension ‐ given that household electricity coverage in Shandong Province has reached 100% [100], the electricity indicator was removed. Furthermore, sanitation and housing indicators were omitted due to data unavailability post-2016 in the CFPS. These were replaced with homeownership and housing area, which are commonly used indicators in multidimensional poverty assessments [90, 101, 102]. (3) Income ‐ the inclusion of income as a dimension in the MPI is a debated topic. Sen [17–19] argued that poverty is a lack of capability, and income is only a means to achieve capability rather than a capability in itself. Consequently, most multidimensional poverty indices do not include income [19, 103]. However, income, while insufficient as a sole measure of poverty, is acknowledged as a significant means to access valuable resources and opportunities [80]. Recognizing this, some international organizations and governments, such as the World Bank [104] and the government of Malaysia [105], incorporated income as a dimension in their poverty assessments. Given the availability of income data in the CFPS and its utility in complementing non-income data, this study included income in the MPI [81, 83]. These dimensions and indicators are shown in Table 2.

**Table 2:** Dimensions, indicators, deprivation cutoffs, and weight settings in this study.

Dimension	Indicator	Deprivation Cutoff	Weight
**Income**	Household Per Capita Income	Household per capita income last year was lower than the minimum living allowance of Shandong Province for that year. ^1^	1/4
**Health**	Body Mass Index (BMI)	Any adult household member with a BMI less than 18 or greater than or equal to 24. ^2^	1/12
	Self-rated Health	Self-rated health status of any household member is poor.	1/12
	Medical Insurance	Any household member does not have medical insurance.	1/12
**Education**	Education Level	Any household member aged 18 or older has an education level below lower secondary education. ^3^	1/8
	School Attendance	Any child between the ages of 6 and 16 is not attending school.	1/8
**Standard of Living**	Cooking Fuel	The household does not use clean fuel such as electricity, liquified petroleum gas, or natural gas for cooking.	1/20
	Drinking Water	The household has no clean water such as tap water, barreled water, purified water, or filtered water at home.	1/20
	Homeownership	The household has no homeownership.	1/20
	Housing Area	The living area per capita is less than 15 square meters. ^4^	1/20
	Household Assets	The total value of all durable goods in the household is no more than 1000 CNY (147 USD). ^5^	1/20

^1^—The annual minimum living allowance in Shandong Province is used as the urban poverty line, which was 3480 CNY (512 USD) per capita in 2010, 4440 CNY (653 USD) per capita in 2012, 5424 CNY (798 USD) per capita in 2014, 5964 CNY (877 USD) per capita in 2016, and 6192 CNY (911 USD) per capita in 2018 [103, 106].

^2^—The standard weight for adults, as set by the National Health Commission of China in Criteria of Weight for Adults, is 18.5 ≤ BMI < 24.0 [107].

^3^—China employs a 9-year compulsory education. The standard for 9-year compulsory education is completing lower secondary education.

^4^—According to Jinan Daily [108], the standard of housing poverty in Shandong Province is 15 square meters per capita. Therefore, households with a living area of no more than 15 square meters per capita are considered poor in housing.

^5^—CFPS defines durable goods as “products with a price of more than 1000 CNY (147 USD) and a natural service life of more than two years” [90]. Most studies in China consider “not owning more than one durable good” as deprivation. Therefore, the threshold was set as 1000 CNY (147 USD).

According to the UNDP and the Oxford Poverty and Human Development Initiative (OPHI), it is crucial for multidimensional poverty measures to be robust. This robustness entails that the measures should not be excessively sensitive to minor modifications in the indicators, cutoffs, or weights used in their calculation [21]. This study utilized the Kendall rank correlation coefficient to assess the robustness of the modified indicators used. Three alternative sets of indicators were tested, with each set excluding school attendance, income, and durable goods from the Global MPI [93, 109]. Given the lack of specific county location information in the China Family Panel Studies (CFPS) data for 2018, this study employed a method of random subdivision of the entire sample into four equal-sized groups.

The Kendall rank correlation coefficients test was employed to examine the correlations between the *M*_*0*_ of the four groups. The results, presented in Table 3, indicated that all Kendall rank correlation coefficients exceed 0.6, with certain correlations displaying statistical significance at the 0.01 level, suggesting a strong relationship among the four sets of indicators. This finding substantiated the robustness of the indicators employed in this study, affirming their reliability in the measurement of multidimensional poverty.

**Table 3:** Kendall rank correlation coefficients for MPI with different indicators in 2018.

	Baseline MPI	School Attendance Removed	Income Removed	Durable Goods Removed
**Baseline MPI**	1.000			
**School attendance removed**	0.667	1.000		
**Income removed**	0.667	1.000 **	1.000	
**Durable goods removed**	1.000 **	0.667	0.667	1.000

Note: ** indicates a significant correlation at the 0.01 level (2-tailed).

### 3.4. Deprivation thresholds

The deprivation threshold is the minimum achievement required for non-deprivation in each indicator [64]. This study mainly referred to the Global MPI of the UNDP [94] and revised its deprivation thresholds according to the national conditions of China and the average living standards of Shandong Province [110]. In addressing the absence of an official urban poverty line in China [9], this study adopted the annual urban minimum living allowance in Shandong Province as the benchmark for the income threshold. This approach aligns with the regional economic context and provides a locally relevant measure of poverty. For the nutrition threshold, the study referred to the weight criteria for adults as set forth by the National Health Commission of China [107]. This criterion offers a standardized, health-based measure to assess nutritional status, thus aiding in the identification of deprivation in this dimension. Regarding the education threshold, the study recognized the implementation of the 9-year compulsory education system in China since 1986 [111]. Consequently, the threshold for education deprivation was set at a level below lower secondary education. This criterion reflects the national education policy and the importance of a minimum level of education in assessing poverty. These various thresholds, crucial for determining deprivation in their respective dimensions, are systematically presented in Table 2 of the study. This tabulation aids in providing a clear and concise reference for the poverty thresholds applied across the different dimensions assessed in this research.

### 3.5. Poverty thresholds

The poverty cutoff *k* determines when a household has enough deprivations to be considered poor [64]. The value of *k* lies between 0 and 1 [21]. Usually, the higher the value of *k*, the higher the average deprivation score and the lower the incidence of multidimensional poverty [1, 81]. However, current studies have no unified standard for the value of *k*; many researchers follow the UNDP (2010) and set the poverty cutoff *k* to 1/3 [23, 64, 93]. That is, if a household’s deprivation score is greater than or equal to 1/3, it will be considered multidimensionally poor.

This study tested multidimensional urban poverty in Shandong Province using poverty cutoff *k* values varying from 0.1 to 0.7 and discovered that when the value of *k* was set between 0.2 and 0.3, both the incidence and the adjusted headcount ratio (*M*_*0*_) experienced a significant decrease compared with the previous or latter values. The results are shown in Tables 4 and 5. This finding is consistent with Alkire and Fang [23], Chi et al. [96], and Huo and Lin [112]. In addition, according to the UNDP and OPHI, the value of *k* is equivalent to the households being deprived in at least one dimension [21, 104, 113, 114]. Therefore, setting the value of *k* to 0.25, which falls between 0.2 and 0.3 and corresponds to one dimension of deprivation, is more feasible for studying multidimensional urban poverty.

**Table 4:** Censored headcount ratio (*H*) with different *k* values.

*K*	2010	2012	2014	2016	2018
**0.1**	81.35%	82.26%	78.75%	74.40%	75.00%
**0.2**	61.90%	67.34%	63.33%	56.31%	58.43%
**0.3**	33.33%	45.56%	37.08%	26.28%	21.39%
**0.4**	21.83%	26.61%	24.17%	15.70%	10.84%
**0.5**	14.29%	17.34%	16.25%	10.92%	7.23%
**0.6**	5.16%	7.66%	5.83%	5.46%	2.11%
**0.7**	0.00%	1.61%	1.25%	1.37%	0.00%

**Table 5:** Adjusted headcount ratio (*M*_*0*_) with different *k* values.

*K*	2010	2012	2014	2016	2018
**0.1**	0.2567	0.2862	0.2637	0.2208	0.2043
**0.2**	0.2266	0.2643	0.2421	0.1952	0.1817
**0.3**	0.1579	0.2109	0.1764	0.1234	0.0941
**0.4**	0.1184	0.1447	0.1321	0.0868	0.0576
**0.5**	0.0841	0.1034	0.0963	0.0653	0.0416
**0.6**	0.0337	0.0514	0.0399	0.0363	0.0139
**0.7**	0.0000	0.0119	0.0094	0.0104	0.0000

**Fig 2:**
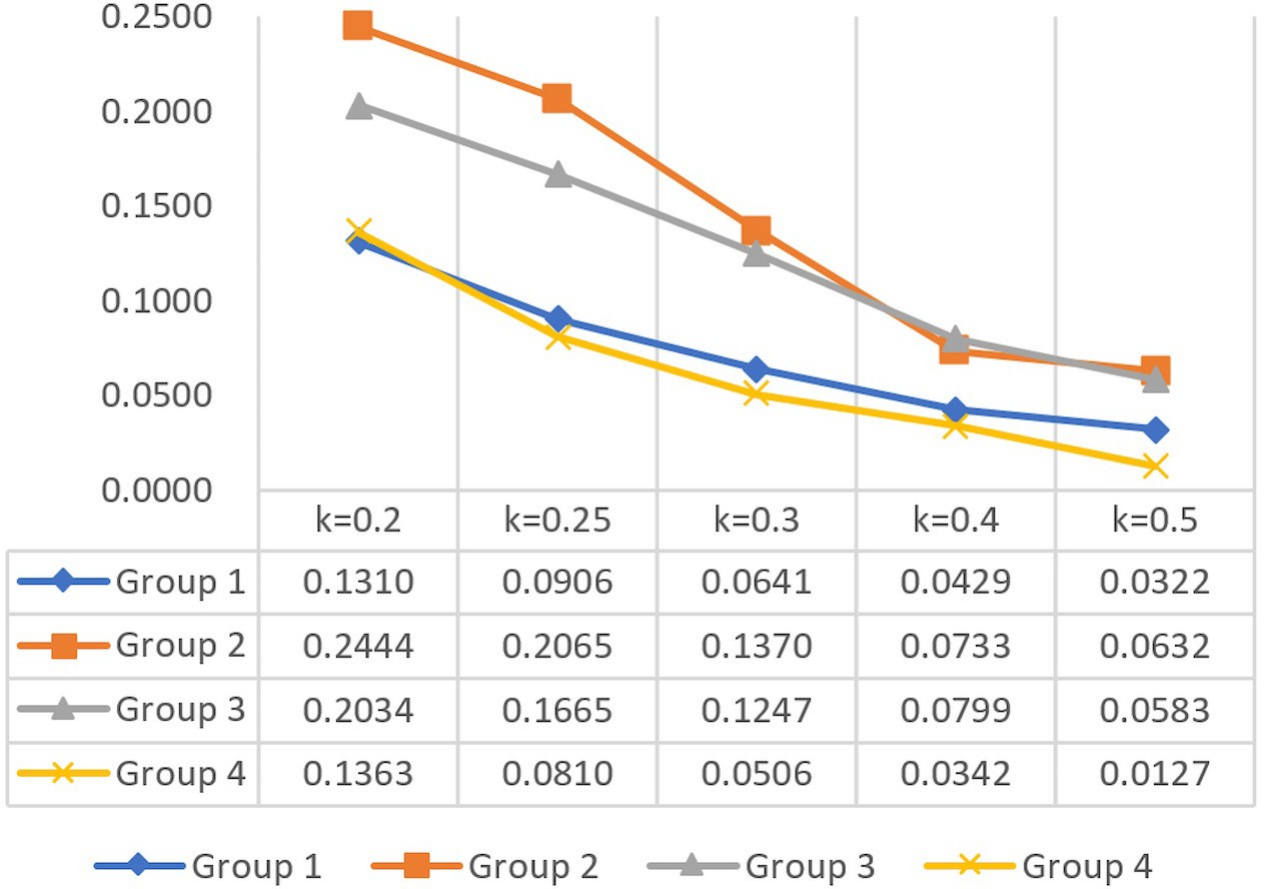
Robustness to the cutoff *k* for different groups. This study employed a dominance analysis to test the robustness of the poverty cutoff. Samples were divided into four random groups. The x-axis represents different *k* values, while the y-axis represents *M*_*0*_ values for different groups. The line graph confirms *M*_*0*_ stability across almost all *k* values, validating the robustness of the poverty cutoff.

To test the robustness of the poverty cutoff, this study referred to Alkire and Santos [93, 109] and UNDP and OPHI [21] and examined the *M*_*0*_ of all samples in CFPS 2018 using a reasonable range of cutoff values between 0.2 and 0.5. This is because, in this study, when *k* is less than 0.2, the majority of respondent households fall into poverty, whereas when *k* is greater than 0.5, barely any households fall into poverty. This study employed a basic dominance analysis; i.e., if group A’s *M*_*0*_ is greater or equal to group B’s *M*_*0*_ for all *k* values, and is strictly greater for at least one *k* value, then group B has lower poverty than group A regardless of the *k* value [109]. This study randomly divided all samples into four equal-sized subgroups. Results in Fig 2 show that Group 1 and Group 4 are less poor than Group 2 and Group 3 for all *k* values, Group 4 is less poor than all other groups for all *k* values except for when *k* = 0.2, and Group 2 is poorer than any other groups except when *k* = 0.4. The ranking of all four groups is stable in almost all cases regardless of the *k* value, particularly between 0.25 and 0.3, proving the robustness of the poverty cutoff chosen in this study.

### 3.6. Weight settings

There are mainly three types of approaches to set weights: normative, data-driven, and hybrid weighting [42]. Normative weighting includes expert opinion, equal weights, and arbitrary weights. Data-driven weighting includes frequency-based, statistical, and most favorable weights. This study referred to previous studies on multidimensional poverty and set equal weights on the dimensions and indicators [23, 47, 64, 76].

To test the robustness of weight settings in multidimensional poverty measurement, this study followed Alkire and Santos [93, 109] and estimated the *M*_*0*_ of all samples in CFPS 2018 using four alternative weighting structures, assigning 50% of the weights to one of the four dimensions and 16.67% to each of the other three dimensions in turn. The weighting schemes are shown in Table 6. Because CFPS does not provide county location information, this study randomly divided the data into four equal-sized subgroups and calculated the *M*_*0*_ of each subgroup. Results in Table 6 showed that all the Kendall rank correlation coefficients for the *M*_*0*_ are greater than 0.6, and some correlations are statistically significant at the 0.01 level. This indicates that the correlation among the five weight settings is very high. Therefore, the robustness of the equal weighting scheme is validated.

**Table 6:** Kendall rank correlation coefficients for MPI with different weights in 2018.

	Equal Weight	50% Income	50% Health	50% Education	50% Standard of Living
**Equal weight**	1.000				
**50% income**	1.000 **	1.000			
**50% health**	0.667	0.667	1.000		
**50% education**	0.667	0.667	1.000 **	1.000	
**50% standard of living**	1.000 **	1.000 **	0.667	0.667	1.000

Note: ** indicates a significant correlation at the 0.01 level (2-tailed).

In addition, since this study disaggregated multidimensional poverty by indicators and compared them cross-sectionally and longitudinally, the setting of weights has no significant impact on the cross-sectional and longitudinal studies [23]. Therefore, equal weights were assigned to each dimension and the respective indicators within each dimension. The weight settings are shown in Table 2.

### 3.7. Determinants of multidimensional urban poverty

This study employed logistic regression to discover the main determinants of multidimensional urban poverty in Shandong Province. Logistic regression analysis was conducted using SPSS Version 22. The equation for logistic regression can be expressed as:

$$ln\left(\frac{p_{j}}{1-p_{j}}\right)=\beta_{0}+\beta_{1}X_{1}+\beta_{2}X_{2}+\cdots +\beta_{n}X_{n}$$

In the equation, *p*_*j*_ denotes the probability that household *j* fells into poverty, *β*_0_ represents the general intercept, *β*_1_, *β*_2_, *β*_*n*_ are the regression coefficients, and *X*_1_, *X*_2_, … *X*_*n*_ denotes the independent variables.

The dependent variable is a dichotomous variable representing whether a household is poor. If a household’s weighted deprivation score is greater than the poverty threshold (*k* = 0.25), the value of the dependent variable is 1; otherwise, it is 0. The weighted deprivation score was calculated using the Dual Cutoff method created by Alkire and Foster [78].

The independent variables include a series of individual-level and household-level variables: (1) Income and employment: household per capita income, household head’s employment status, and government subsidy. (2) Human capital: household head’s education level, household member’s health, and household member’s medical insurance coverage. (3) Individual characteristics: household head’s gender, age, and marital status. (4) Household structure: household size, aged-dependency ratio, and youth dependency ratio. (5) Household asset ownership: home ownership, car ownership, living area per capita, and durable goods. (6) Household infrastructure accessibility: drinking water and cooking fuel accessibility.

## 4. Results

### 4.1. Bias test

This study analyzed urban poverty using CFPS, a secondary data source; there may be bias in the questionnaire design, sampling, or data collection process [115]. Therefore, the following bias tests were conducted to increase the reliability of multidimensional poverty measurement methods.

#### 4.1.1 Non-response bias test

According to Compton et al. [116], non-response bias occurs when survey analysis is limited to data from respondents who differ from non-respondents, and non-response bias implies that the demographics of the sample are different from those of the population. Because the CFPS data are secondary, this study cannot modify its questionnaire questions during the survey’s development; the best way to identify non-response bias is to compare sample estimates to the population values [117].

The response rate for the CFPS baseline survey in 2010 was 81.25%, and four rounds of follow-up surveys on the full sample were conducted. To address common method bias, this study compared the 2018 population data of Shandong Province with the 2018 CFPS data. The urban per capita income of Shandong Province in 2018 was 36,789 CNY, compared to 31,429 CNY in CFPS. In 2018, 14.38% of Shandong Province had a college education, 14.33% had an upper secondary education, 14.33% had a lower secondary education, and 23.69% had an elementary education. The corresponding percentages in CFPS were 19.60%, 14.33%, 33.40%, and 19.60%. The gender ratio of Shandong Province in 2018 was 1.03, while in the CFPS, it was 1.02. The youth dependency ratio of Shandong Province was 17.9%, and the aged-dependency ratio was 14.9%, while those in the CFPS were 18.78 and 14.04, respectively. In Shandong Province in 2018, 93.9% of urban residents had health insurance, compared to 94% in the CFPS. The housing area per capita in Shandong Province was 43.2, compared to 48.08 in CFPS. Approximately 82.8% of households in Shandong Province had access to tap water, compared to 85.8 percent in CFPS. The use of clean energy, such as natural gas or electricity as cooking fuel, was 87.8% in Shandong Province, compared to 89.5% in CFPS. This study employed the Wilcoxon signed-rank test to examine if there were statistically significant differences between the population data of Shandong Province and CFPS data. According to the results, the median difference was −0.5, z = −0.35, and *p* = 0.972. It can be concluded that there are no significant differences between the population data of Shandong Province and CFPS data.

#### 4.1.2 Common method bias test

Common method bias is usually caused by a single measurement and data from the same source, i.e., the same person in the same measurement using the same questions or questions with similar characteristics [118, 119]. Because most CFPS survey data were self-reported, common method bias had to be addressed. This study used Harman’s single-factor test to examine the independent variables (see Table 2) in CFPS data from 2010 to 2018 in Shandong Province. The results showed that the first principal factor explained 19.55% of the variance in 2010, 20.41% in 2012, 21.87% in 2014, and 17.74% in 2018. Because there was no school attendance poverty in the 2016 CFPS data, this study excluded this variable and tested other variables using Harman’s single factor test, and the results showed that the first principal factor value was 24.43%. The results of all Harman’s single-factor tests from 2010 to 2018 were lower than 40%, indicating no serious common method bias in this study [119–121].

### 4.2. Changes in multidimensional urban poverty

Table 7 shows the results of the multidimensional urban poverty changes in Shandong Province from 2010 to 2018. The incidence of multidimensional urban poverty (*H*) in Shandong Province decreased from 47.62% in 2010 to 36.45% in 2018. The adjusted headcount ratio (*M*_*0*_) in Shandong Province fell from 0.1965 in 2010 to 0.1358 in 2018. However, compared with the poverty incidence, the intensity of multidimensional urban poverty in Shandong Province only decreased from 41.27% in 2010 to 37.25% in 2018. The intensity of urban poverty in Shandong Province did not decrease correspondingly with the decline in the poverty incidence rate from 2010 to 2018, indicating deficiencies in urban poverty alleviation efforts in Shandong Province.

**Table 7:** Changes in multidimensional urban poverty from 2010 to 2018 (*k* = 0.25).

Year	*H*	*A*	*M*_0_	Margin of Error
**2010**	47.62%	41.27%	0.1965	6.17
**2012**	57.26%	42.46%	0.2431	6.22
**2014**	52.50%	41.75%	0.2192	6.33
**2016**	39.25%	40.55%	0.1592	5.73
**2018**	36.45%	37.25%	0.1358	5.38

To analyze changes in the incidence of poverty in each indicator, this study calculated the censored headcount ratio for each indicator from 2010 to 2018. Table 8 shows that the censored headcount ratios of income, education level, medical insurance, cooking fuel, and drinking water decreased significantly from 2010 to 2018. This demonstrates that Shandong Province has made remarkable achievements in increasing urban income, improving education levels, expanding medical insurance coverage, and developing clean drinking water and energy supply. The censored headcount ratio of BMI and self-rated health sustained a high level without a significant decrease from 2010 to 2018. Shandong Province should invest more efforts in these aspects to improve the health conditions of urban households in the future. The censored headcount ratio of homeownership, housing area, and school attendance remained very low from 2010 to 2018. Note that CFPS only collected data on TV ownership in durable goods in 2010, resulting in the low censored headcount ratio of household assets in 2010.

**Table 8:** Changes in censored headcount ratio for each indicator from 2010 to 2018.

Indicators	2010	2012	2014	2016	2018
**Income**	23.41%	29.03%	28.75%	12.97%	10.54%
**Body Mass Index**	36.11%	43.95%	43.33%	36.18%	32.83%
**Self-rated Health**	19.05%	30.65%	19.58%	19.80%	18.67%
**Medical Insurance**	11.90%	14.92%	10.00%	8.87%	7.83%
**Education Level**	43.25%	47.18%	45.83%	36.18%	32.83%
**School Attendance**	0.40%	1.21%	0.42%	0.00%	0.30%
**Cooking Fuel**	28.57%	25.81%	17.50%	15.02%	8.73%
**Drinking Water**	16.67%	17.34%	18.33%	12.63%	11.14%
**Homeownership**	5.16%	12.10%	7.92%	8.19%	5.12%
**Housing Area**	3.57%	3.23%	3.75%	3.41%	3.92%
**Household Assets**	1.19%	12.50%	10.00%	15.70%	8.13%

### 4.3. Contribution of the indicators to multidimensional urban poverty

In order to understand the contribution of each indicator to multidimensional urban poverty, this study disaggregated the adjusted headcount ratio (*M*_*0*_) by different indicators. As depicted in Table 9, from 2010 to 2018, education level and income contributed the most to multidimensional urban poverty, followed by BMI and self-rated health. Cooking fuel, medical insurance, drinking water, household assets, and homeownership contributed relatively little to multidimensional urban poverty, while housing area and school attendance contributed the least. Regarding the changes in the contribution of each indicator, the contribution of income to urban poverty decreased significantly from 29.78% in 2010 to 19.41% in 2018. From 2010 to 2018, the contribution of BMI, self-rated health, and education level to urban poverty increased by 4.84%, 3.38%, and 2.72%, respectively. However, their censored headcount ratios decreased during the same period. The contribution of cooking fuel decreased from 7.27% in 2010 to 3.22% in 2018. The contribution of other indicators did not change significantly.

**Table 9:** Contribution of the indicators to multidimensional urban poverty from 2010 to 2018.

Indicators	2010	2012	2014	2016	2018
**Income**	29.78%	29.85%	32.79%	20.37%	19.41%
**Body Mass Index**	15.31%	15.07%	16.47%	18.94%	20.15%
**Self-rated Health**	8.08%	10.50%	7.45%	10.36%	11.46%
**Medical Insurance**	5.05%	5.11%	3.80%	4.65%	4.81%
**Education Level**	27.51%	24.26%	26.14%	28.41%	30.23%
**School Attendance**	0.25%	0.62%	0.24%	0.00%	0.28%
**Cooking Fuel**	7.27%	5.31%	3.99%	4.72%	3.22%
**Drinking Water**	4.24%	3.57%	4.18%	3.97%	4.10%
**Homeownership**	1.31%	2.49%	1.81%	2.57%	1.89%
**Housing Area**	0.91%	0.66%	0.86%	1.07%	1.44%
**Household Assets**	0.30%	2.57%	2.28%	4.93%	3.00%

### 4.4. Determinants of multidimensional urban poverty

#### 4.4.1. Descriptive analysis

Table 10 shows the frequency, percentage, mean, and standard deviation of the variables. In 2018, 54.5% of household heads were male, while 45.2% were female. The average age of household heads was 49.35 years. The proportion of unmarried household heads was 5.7% of married household heads was 87.0% of divorced household heads was 0.9%, and of widowed household heads was 6.3%. The average household size was 3.11, slightly higher than the average level in Shandong Province [103]. The aged-dependency ratio was 18.78%, and the youth dependency ratio was 14.04%. Some 12.30% of household heads had no education, while 19.60%, 33.40%, 15.10%, and 19.60% had elementary, lower secondary, upper secondary, and college education, respectively. In 2018, 70.20% of household heads were employed, 1.80% were unemployed, and 24.70% were retired. The unemployment rate was relatively low.

**Table 10:** Descriptive analysis of multidimensional urban poverty in 2018.

Variables	Frequency ^1^	Percentage	Mean	Standard Deviation
**Per capita income (CNY)**			31,426.48	37,288.04
**No education (1 = yes; 0 = no)**	41	12.30%	0.12	0.329
**Elementary education (1 = yes; 0 = no)**	65	19.60%	0.20	0.397
**Lower secondary education (1 = yes; 0 = no)**	111	33.40%	0.33	0.472
**Upper secondary education (1 = yes; 0 = no)**	50	15.10%	0.15	0.358
**College or above (1 = yes; 0 = no)**	65	19.60%	0.20	0.397
**Gender (1 = male; 0 = female)**	181 (Male)	54.50% (Male)	0.55	0.499
**Unmarried (1 = yes; 0 = no)**	19	5.70%	0.06	0.233
**Married (1 = yes; 0 = no)**	289	87.00%	0.87	0.336
**Divorced (1 = yes; 0 = no)**	3	0.90%	0.01	0.095
**Widowed (1 = yes; 0 = no)**	21	6.30%	0.06	0.244
**Age (years)**			49.35	15.584
**Employed (1 = yes; 0 = no)**	233	70.20%	0.73	0.447
**Unemployed (1 = yes; 0 = no)**	6	1.80%	0.02	0.136
**Retired (1 = yes; 0 = no)**	82	24.70%	0.26	0.437
**Government subsidized (1 = yes; 0 = no)**	56	16.90%	0.17	0.375
**Aged-dependency ratio** ^**2**^			18.78%	34.45%
**Youth dependency ratio** ^**3**^			14.04%	17.48%
**Household size**			3.11	1.478
**Household member unhealthy (1 = yes; 0 = no)**	86	25.90%	0.26	0.439
**All household members have medical insurance (0 = yes; 1 = no)**	33	9.90%	0.12	0.328
**Household has car ownership (0 = yes; 1 = no)**	193	58.10%	0.58	0.494
**Household has house ownership (0 = yes; 1 = no)**	54	16.30%	0.16	0.370
**Living area per capita (m** ^**2**^ **)**			48.08	35.032
**Household has clean drinking water (0 = yes; 1 = no)**	47	14.20%	0.14	0.349
**Household has clean cooking fuel (0 = yes; 1 = no)**	35	10.50%	0.11	0.308
**Household owns more than one durable good (0 = yes; 1 = no)**	33	9.90%	0.10	0.300

^1^—The frequency and percentage apply when the variable equals 1.

^2^—The aged-dependency ratio in this study refers to the number of individuals aged 65 or older divided by household size [48, 122].

^3^—The youth dependency ratio in this study refers to the number of individuals aged 0 to 14 divided by household size [48, 122].

#### 4.4.2. Regression analysis

As shown in Table 11, the logit model in this study fitted the data very well. The significance level of Omnibus Tests of Model Coefficients is less than 0.001, and Chi-square is 260.370 with 24 degrees of freedom, which indicates the significance level of the model. The value of −2 Log likelihood is 151.265; Cox and Snell R Square is 0.565; and Nagelkerke R Square is 0.772. The significance level of the Hosmer and Lemeshow Test is 0.064. And the model correctly predicted 89.1% of the observations.

**Table 11:** Classification table.

		Predicted		
		Poverty		Percentage Correct
		No	Yes	
**Poverty**	No	183	15	92.4
	Yes	19	96	83.5
**Overall Percentage**				89.1

Note: The cut value is 0.500.

Due to the large number of explanatory variables in the logit model, this research employed a Variance Inflation Factor (VIF) test to determine whether or not the regression model had a collinearity issue. As shown in Table 12, the results indicated that all VIF values were less than 4. According to Akinwande et al. [123], Hair et al. [124], James et al. [125], and O’brien [126], a VIF value less than 5 indicates that there were no significant collinearity issues among the variables. Therefore, this regression model had no serious collinearity issue.

**Table 12:** Independent variables in the model.

Variables	Coef.	Standard Error	z	*p*-Value	Odds Ratio	VIF
**Per capita income**	−0.276	0.101	7.483	0.006 ***	0.759	1.575
**No education**	0.363	1.038	0.122	0.727	1.437	2.407
**Elementary education**	1.974	0.908	4.729	0.030 **	7.197	2.393
**Lower secondary education**	−1.154	0.844	1.870	0.172	0.315	2.455
**Upper secondary education**	−0.300	0.824	0.132	0.716	0.741	1.687
**Gender**	0.580	0.473	1.504	0.220	1.786	1.330
**Unmarried**	−1.514	1.582	0.917	0.338	0.220	2.579
**Married**	0.475	1.071	0.196	0.658	1.607	2.535
**Divorced**	−20.350	19,511.256	0.000	0.999	0.000	1.231
**Age**	0.0026	0.027	0.933	0.334	1.027	3.702
**Employed**	0.0045	0.578	0.006	0.938	1.046	1.487
**Unemployed**	−24.783	15,889.511	0.000	0.999	0.000	1.204
**Government subsidized**	−0.497	0.625	0.632	0.427	0.608	1.206
**Aged-dependency ratio**	2.279	0.893	6.518	0.011 **	9.767	2.491
**Youth dependency ratio**	0.412	1.742	0.056	0.813	1.509	2.178
**Household size**	0.614	0.248	6.155	0.013 **	1.849	2.241
**Household member unhealthy**	3.431	0.576	35.480	0.000 ***	30.902	1.154
**All household members have medical insurance**	3.505	0.714	24.098	0.000 ***	33.280	1.108
**Household has car ownership**	0.600	0.574	1.092	0.296	1.822	1.664
**Household has homeownership**	1.509	0.760	3.946	0.047 **	4.521	1.312
**Living area per capita**	0.001	0.006	0.036	0.850	1.001	1.318
**Household has clean drinking water**	3.610	0.732	24.295	0.000 ***	36.970	1.265
**Household has clean cooking fuel**	1.797	0.821	4.791	0.029 **	6.033	1.316
**Household owns more than one durable good**	2.412	0.885	7.427	0.006 ***	11.156	1.206
**Constant**	−6.729	2.268	8.806	0.003	0.001	

Note: *** and ** denote significance levels at 0.01 and 0.05, respectively.

Logistic regression results in Table 12 show that the significance levels for per capita income, household members’ health, medical insurance, drinking water, and durable goods had the most significant impact on multidimensional urban poverty in Shandong Province, with significance levels below 0.01. Elementary education, aged-dependency ratio, household size, homeownership, and cooking fuel also had a significant effect on multidimensional urban poverty, with significance levels less than 0.05.

For Hypothesis 1, it was discovered that per capita income (OR = 0.759, *p* = 0.01) had a significant negative effect on multidimensional urban poverty. In order to measure the relationship between income and multidimensional poverty more accurately, the per capita income was divided into ten deciles [14]. The odds ratio for per capita income is 0.759, which means with each increase in per capita income, the likelihood of a household falling into poverty decreases by 24.1%. This finding validated the effectiveness of increasing household income in alleviating urban poverty.

For Hypothesis 2, it was found that the health status of household members (OR = 30.902, *p* = 0.01) and possession of medical insurance (OR = 33.280, *p* = 0.01) were significantly inversely related to multidimensional urban poverty. Households with unhealthy members were 30.902 times more likely to be poor than healthy households. Households, where not all members had medical insurance were 33.28 times more likely to be poor compared to those where all members had medical insurance.

For Hypothesis 3, this study discovered that household heads with no education (OR = 1.437) or elementary education (OR = 7.197, *p* = 0.05) were more likely to be poor than other education levels. Among these, household heads with elementary education were 7.197 times more likely to be poor than those without. In contrast, household heads with lower secondary education (OR = 0.315) and upper secondary education (OR = 0.741) were less likely to be poor, although these findings are not statistically significant.

For Hypothesis 4, household size (OR = 1.849, *p* = 0.05) and aged-dependency ratio (OR = 9.767, *p* = 0.05) also had significant positive impacts on multidimensional urban poverty. Households with larger household sizes were 1.849 times more likely to be poor than those with smaller household sizes. Households with higher aged-dependency ratios were 9.767 more likely to be poor than those with lower aged-dependency ratios.

For Hypothesis 5, infrastructure accessibility, particularly access to drinking water (OR = 36.970, *p* = 0.01) and cooking fuel (OR = 6.033, *p* = 0.05), showed a significant negative association with multidimensional urban poverty. Households without clean drinking water were 36.97 times more likely to be poor than those with clean drinking water. Households without clean cooking fuel were 6.033 times more likely to be poor than those with cooking fuel.

For Hypothesis 6, household assets, including durable goods (OR = 11.156, *p* = 0.01) and homeownership (OR = 4.521, *p* = 0.05), also had significant negative effects on multidimensional urban poverty. Households with less than one durable good were 11.156 times more likely to be poor than those with more than one durable good. Households without homeownership were 4.521 times more likely to be poor than those with homeownership.

## 5. Discussion

This study, through its detailed measurement of multidimensional urban poverty, has yielded several notable findings regarding the dynamics of urban poverty in Shandong Province, China, from 2010 to 2018. Furthermore, the study identified education level, income, BMI, and self-rated health as the main contributors to multidimensional urban poverty. Regression analysis indicated that income, health status, access to clean drinking water, and ownership of durable goods emerged as key determinants of multidimensional urban poverty in Shandong Province.

### 5.1. Changes in multidimensional urban poverty

The analysis of changes in multidimensional urban poverty within Shandong Province revealed a significant disconnect between the incidence and intensity of poverty from 2010 to 2018. Notably, while there was a decrease in the number of households experiencing poverty (incidence), the depth or severity of poverty among those who remained impoverished (intensity) did not witness a corresponding reduction. This pattern suggested that the anti-poverty strategies were primarily effective in elevating households that were marginally poor above the poverty threshold. However, these measures fell short in adequately supporting the poorest households, which continued to face profound deprivation. This phenomenon observed in Shandong Province aligns with findings from other studies, such as those by Yu [78] and Zhang et al. [127]. These researchers also identified a similar disparity in the changes between the incidence and intensity of multidimensional poverty. The persistence of high-intensity poverty, despite a reduction in its incidence, raised concerns about the effectiveness of poverty alleviation strategies in reaching the most disadvantaged segments of the population. It became evident that for Shandong Province to align with the United Nations SDGs, particularly the goal of "leaving no one behind," there is a need for more concerted and targeted efforts in poverty alleviation. The focus should shift towards intensifying support for households experiencing deep poverty. By ensuring that these households benefit substantially from poverty reduction programs, Shandong Province can move towards a more inclusive and equitable approach to combating poverty, ultimately working towards the eradication of poverty in all its forms and dimensions.

### 5.2. Contribution of each indicator to multidimensional urban poverty

The decrease in the contribution of income was mainly due to the significant decrease in the censored headcount ratio of income from 2010 to 2018. Despite the increase in BMI, self-rated health, and education level in their contribution, during the same period, their censored headcount ratios decreased by 3.28%, 0.38%, and 10.42%, respectively. This contrast can be attributed to the significant decrease in the censored headcount ratio of other indicators, such as income and cooking fuel, from 2010 to 2018, resulting in a more prominent contribution of BMI, self-rated health, and education level to urban poverty in comparison. This indicated the urgency of urban anti-poverty efforts in health and education in Shandong Province. Addressing poverty in these aspects should be a higher priority for future urban anti-poverty efforts in Shandong Province. The decrease in the contribution of cooking fuel was mainly due to the dramatic decline in its censored headcount ratio from 2010 to 2018. Shandong Province should invest less in other aspects of future urban anti-poverty efforts.

### 5.3. Determinants of multidimensional urban poverty

This study validated Hypothesis 1 by showing that household income had a significant negative effect on urban poverty. Although there are debates about whether income should be considered a component of the MPI, according to Sen’s capability approach, it is considered a vital resource that enables individuals to access valuable ends, such as meeting basic human needs and thereby enhancing their capabilities [22, 80]. Therefore, this study incorporated it into the MPI. According to this finding, Shandong Province should make greater efforts to boost the income of urban poor households, including expanding income sources, providing vocational training, and increasing financial subsidies. This finding is consistent with studies by Wang [8], Xiao et al. [81], and Javed and Asif [128], which all indicate a negative correlation between income and multidimensional poverty.

This study validated Hypothesis 2 by demonstrating that the health of household members had a significant negative impact on urban poverty. Health plays an important role in Sen’s capability approach. It can be viewed as functionings, representing a state of well-being, and as a capability, indicating the opportunity to attain a certain level of health [88]. It can also be considered a personal conversion factor, influencing how individuals transform resources into capability [129]. According to the National Health Commission of China, about 40% of poor households in China fell into poverty or returned to poverty due to illness [82]. The good health of household members can provide a stable source of income or reduce the household’s additional expenses, thereby lowering the risk that the household falls into poverty [84, 85]. This finding is consistent with studies conducted by Gu et al. [84], Guo & Zhou [47], Mohanty et al. [99], Moyo et al. [130], Yu [80], Xie & Che [85], and Zhong & Lin [102], which all indicated that household heads with severe illnesses are more likely to have reduced productivity, employment opportunities, and income, increasing their risk of falling into multidimensional poverty.

This study supported Hypothesis 3 by demonstrating a strong correlation between limited education and urban poverty. The results showed that household heads with education levels higher than elementary school were less likely to fall into multidimensional poverty compared to those with lower education levels. Similar to health, education plays a crucial role within the framework of the capability approach, functioning both as a capability and a functioning. And as a conversion factor, it influences an individual’s ability to convert means into well-being [131]. This finding is consistent with Adepoju & Oyewole [132], Artha & Dartanto [133], Fahad et al. [134], Najitama et al. [135], Tang et al. [86], and Zhong & Lin [102], who all agreed that education empowers individuals by providing essential knowledge and skills, enhancing job opportunities and earning potential [133]. Furthermore, education fosters a healthy lifestyle and knowledge, contributing to improved health and overall quality of life [136]. Therefore, it is a key factor for households to escape poverty.

This research showed a significant positive correlation between household size and the aged-dependency ratio with urban poverty, strongly supporting Hypothesis 4. This finding is consistent with Chen et al. [137], Guo & Zhou [47], Hashmi et al. [138], Oyekale et al. [139], and Tran et al. [48]. Usually, the larger the household size, the higher the household’s expenditure on consumption, and correspondingly, the lower the household’s expenditures on other necessities such as education and health care. However, this contradicts Adepoju & Oyewole [132] and Najitama’s [135] studies on longitudinal or dynamic poverty. They indicated that adding a new household member reduces the likelihood of longitudinal poverty, as a new household member can provide additional labor, resulting in extra income. This study’s findings differ because school-age children are unproductive until they enter the workforce, contributing to household expenses rather than incomes. However, in the long term, investing in children’s education can yield positive outcomes for households [132].

Regarding the aged-dependency ratio, this study’s findings align with those of Alkire and Fang [23], Najitama et al. [135], Wang et al. [79], and Zhong and Lin [102]. They discovered that the elderly contribute little to productivity. As the aged-dependency ratio increases in a household, expenditures on caring for the elderly rise, leading to reduced allocations for other necessities. Also, a high aged-dependency ratio may result in the working-age population reducing their work hours to care for the elderly, thereby decreasing the household’s income. Therefore, it can exacerbate a household’s poverty.

This study supported Hypothesis 5 by showing that infrastructure accessibility, including clean drinking water and cooking fuel, had significant negative effects on urban poverty. Access to clean drinking water and cooking fuel is widely considered a basic human right by international organizations such as the United Nations [140] and the World Bank [141]. It is an essential component of the United Nations SDGs [127]. Studies by Alkire & Fang [23], Fonta et al. [142], Nadeem et al. [143], and Xiao et al. [81] highlight the critical importance of clean drinking water for individual health. Water contamination is a significant contributor to infectious diseases, impacting the health aspect of multidimensional poverty [142–144]. Studies by Guo et al. [145], Jiao [146], Mehra et al. [147], Nadeem et al. [143], and Wang et al. [148] showed a negative correlation between clean cooking fuel and multidimensional poverty. As highlighted by the World Health Organization [149], unclean cooking fuel contributes to indoor air pollution, increasing the risk of respiratory diseases and other health issues, especially for women and children within the household. These health challenges further exacerbate poverty among poor households [133].

This study supported Hypothesis 6 by demonstrating that asset ownership, including durable goods and homeownership, had a significant negative effect on urban poverty. Household assets are the stock of wealth, which reflects the long-term material well-being status of the household [79, 133]. Durable goods, in particular, which offer enduring service through repeated usage, play a crucial role [150]. Studies by Adeboju & Oyewole [132], Dutta & Kumar [151], Qi et al. [152], and Xiao et al. [81] highlighted how these goods elevate household productivity, enable information access, and enhance overall quality of life, elevating the likelihood of escaping poverty. Furthermore, studies by Artha & Dartanto [133], Adeboju & Oyewole [132], and Sevinc [153] suggested that homeownership represents a family’s financial stability and is associated with improving their living conditions. Households lacking housing often struggle to cope with sudden financial challenges or emergencies, making them more vulnerable to poverty.

### 5.4. Research implications

#### 5.4.1. Practical implications

Currently, China has only implemented targeted anti-poverty strategies in rural areas [8]. In 2015, Xi [7] first elaborated on the concept of “targeted poverty alleviation” in 2015 and pointed out that the direction of China’s poverty alleviation efforts should be accurate identification, targeted assistance, and household-specific policies. This should be the future direction of China’s urban poverty alleviation work [10, 154]. This study used a multidimensional poverty measure to measure urban poverty at the household level, which helps to better target urban poor households and help the government implement household-specific, targeted poverty alleviation policies. Also, because the economic development level and regional economic differences of Shandong Province are similar to those of China, this study’s policy recommendations can be generalized to the whole country [55, 155, 156].

#### 5.4.2. Theoretical implications

This study improved existing poverty measurement methods. Poverty measurement is the basis for developing anti-poverty policies [157]. This study attempted to establish an MPI that is more relevant and representative of China and Shandong Province. This study improved the multidimensional poverty measurement methods, including the setting of poverty thresholds and weights, and conducted a few tests to improve the method’s reliability and validity in order to target urban poor households better, thereby providing a theoretical basis for future urban multidimensional poverty research.

## 6. Conclusions

This paper measured multidimensional urban poverty in Shandong Province, China, using the Dual Cutoff method created by Alkire and Foster [78]. The results showed that although the incidence of multidimensional urban poverty in Shandong Province decreased dramatically from 2010 to 2018, the intensity of multidimensional urban poverty did not decrease correspondingly. This indicated that urban poverty standards in Shandong Province were too low from 2010 to 2018, allowing only marginally poor households to escape poverty and leaving the poorest behind [158]. To achieve the goal of “leave no one behind,” Shandong Province should prioritize reducing the intensity of urban poverty rather than its incidence in the future.

This paper disaggregated multidimensional urban poverty in Shandong Province to determine each indicator’s contribution. It was discovered that education level and income contributed the most to urban poverty, followed by BMI and self-rated health. Among these indicators, the contribution of income decreased significantly from 2010 to 2018, mainly due to a significant decline in its censored headcount ratio. The contribution of BMI, self-rated health, and education level increased slightly from 2010 to 2018; however, in contrast, their censored headcount ratio decreased during the same period. This can be attributed to Shandong Province’s achievements of poverty alleviation in other indicators, resulting in a more prominent contribution of these indicators. This indicated the urgency of future urban health and education poverty alleviation efforts in Shandong Province.

This paper also analyzed multidimensional urban poverty in Shandong Province using logistic regression to discover the main determinants of multidimensional urban poverty. The results showed that income, health (including household members’ health status and medical insurance), drinking water, and durable goods were the most significant determinants of multidimensional urban poverty in Shandong Province. Based on these findings, this study presents the following policy recommendations.

Regarding income, the government should provide vocational training for urban poor individuals to create employment opportunities for them. Additionally, it is recommended that the government increase financial subsidies to provide immediate relief for vulnerable households.

In terms of health, it is recommended that the government expand the coverage and reimbursement rates of medical insurance. For patients with serious illnesses, the government can collaborate with insurance companies to introduce affordable private medical insurance, providing insurance coverage for major illnesses.

China has implemented nine years of compulsory education, which includes only elementary and lower secondary education [111]. The government should invest in expanding public kindergartens or providing subsidies to private kindergartens, reducing the burden on poor households. For senior high school and college students, the government should broaden the availability of scholarships and student loans to benefit more urban poor students.

To address the issues of large household size and high aged-dependency ratios, the government can assist by providing basic pensions for urban poor households. It is also recommended that the government invest more in community service facilities, including community-based childcare services and daytime care for the elderly, easing the burden of poor households.

To improve the standard of living of urban households, the government should expand the supply of essential infrastructure, such as clean water and cooking fuel, and provide corresponding financial subsidies to these households. The government should also provide low-rent housing or housing subsidies for urban poor households, ensuring they have access to stable housing.

This research has two major limitations. First, poverty is a complex phenomenon [21, 64], and it is challenging to identify indicators that cover all its aspects [159]. Future studies should tailor the selection of indicators according to the customs and living standards of their respective study areas. Second, this study did not take heterogeneity into account, such as spatial and household heterogeneity. This is because this study aims to understand the overall situation of urban poverty in Shandong Province, including poverty incidence and intensity, rather than focusing on different subgroups. If necessary, future multidimensional poverty studies should consider heterogeneity to explore the spatial and household disparities in multidimensional poverty.

The main contribution of this paper is that it improved the current multidimensional poverty measurement methods, especially on the setting of poverty thresholds and weights. This study also conducted a series of robustness tests to increase the validity and reliability of multidimensional poverty measures. Second, this study discovered that the decrease in multidimensional poverty in urban Shandong Province is mainly due to poverty incidence rather than poverty intensity, indicating the inadequacy of urban anti-poverty efforts in Shandong Province. Moreover, due to the representativeness of Shandong Province in China, this study’s policy recommendations can be generalized to the whole country [55]. Therefore, it has significant implications for future urban anti-poverty efforts in China.

## Supporting information

S1 Dataset Urban household and individual data of Shandong Province in 2010. (XLSX)

S2 Dataset Urban household and individual data of Shandong Province in 2012. (XLSX)

S3 Dataset Urban household and individual data of Shandong Province in 2014. (XLSX)

S4 Dataset Urban household and individual data of Shandong Province in 2016. (XLSX)

S5 Dataset Urban household and individual data of Shandong Province in 2018. (XLSX)
